# Mucosal Grafts and Flaps in Draf IIb and Draf III: A Systematic Review and Meta‐analysis

**DOI:** 10.1002/ohn.1374

**Published:** 2025-08-04

**Authors:** Alexander Lein, David T. Liu, Birgit Knerer, Christian A. Mueller, Nicole Rotter, Erich Vyskocil, Archana Jaiswal, Faris F. Brkic, Rajiv Bhalla

**Affiliations:** ^1^ Department of Otorhinolaryngology–Head and Neck Surgery Medical University of Vienna Vienna Austria; ^2^ Department of Otolaryngology–Head and Neck Surgery Stanford University School of Medicine Palo Alto California USA; ^3^ Department of Otorhinolaryngology–Head and Neck Surgery University Clinic Mannheim, University of Heidelberg Mannheim Germany; ^4^ Department of Otorhinolaryngology Manchester University NHS Foundation Trust Manchester UK; ^5^ Department of Otorhinolaryngology, Manchester Centre for Clinical Neurosciences Northern Care Alliance NHS Foundation Trust Salford UK

**Keywords:** Draf IIb, Draf III, endoscopic surgical procedure, frontal sinus, mucosal flap

## Abstract

**Objective:**

To evaluate the impact of mucosal grafts and flaps on neo‐ostium patency and clinical outcomes in patients undergoing Draf IIb or III frontal sinus drill‐out procedures.

**Data Sources:**

A systematic search was conducted across PubMed, Medline, Cochrane Library, Scopus, and Web of Science databases in accordance with Preferred Reporting Items for Systematic Reviews and Meta‐analyses (PRISMA) guidelines.

**Review Methods:**

Following PRISMA guidelines, consecutive studies were included, and a meta‐analysis was performed. Study characteristics, patient demographics, surgical outcomes, and neo‐ostium patency rates were extracted. Pooled relative risks (RRs) and mean differences (MDs) were estimated using random‐effects models. Publication bias was evaluated via funnel plots and Egger's test.

**Results:**

Nine studies with 375 patients undergoing mucosal reconstruction were included, following 116 (30.9%) Draf IIb and 259 (69.1%) Draf III procedures. Four comparative studies were eligible for meta‐analysis. Draf III was performed in 97.2% of cases (n = 318), with 165 (51.9%) patients in the flap group and 153 (48.1%) in the no‐flap group. Mean follow‐up was 41.3 ± 9.5 months. Mucosal flaps/grafts significantly improved neo‐ostium patency (RR 1.087, 95% CI: 1.018‐1.159, *P* = .011) and reduced loss of neo‐ostium area (MD 19.52%, 95% CI: 9.97‐29.06, *P* < .01). Clinical outcomes, including polyp scores and patient‐reported measures, showed high heterogeneity, limiting synthesis.

**Conclusion:**

Mucosal reconstruction may enhance neo‐ostium patency but could only be analyzed for Draf III. Variability in study design and outcome reporting remains a limitation. Standardized methodologies are essential to accurately assess the role of mucosal grafting in frontal sinus surgery.

Despite prolific advancements in endoscopic sinus surgery, restenosis remains the primary cause of failure in extended frontal sinus procedures.^1^ Draf IIb and Draf III procedures, designed to optimize sinus drainage by enlarging the outflow tract, require resection of the frontal sinus floor, inter‐sinus septum, and nasal septum, often leaving large areas of exposed bone.^2^, ^3^, ^4^ Extensive drilling and a lack of immediate mucosal coverage make the exposed bone susceptible to osteoneogenesis and inflammation, both of which contribute to neo‐ostium restenosis.^5^, ^6^ In particular, restenosis rates for Draf IIb and Draf III are estimated at 13.1% and 17.1%, respectively, with 9.0% of Draf III cases requiring a revision surgery.^1^, ^7^

To mitigate this, mucosal flaps or grafts are harvested intraoperatively and placed over exposed bone.^8^ This concept of mucosal reconstruction dates back to 1935, when Sewall described the use of a medially harvested mucoperiosteal flap during open frontal sinus surgery to preserve the frontal recess.^9^ With the transition to endoscopic techniques, this approach was adapted. To date, mucosal flaps are derived from the nasal septum, lateral nasal wall, or turbinates and are either pedicled (maintaining their blood supply) or free grafts (relying on secondary revascularization).^10^, ^11^, ^12^, ^13^, ^14^ By covering exposed bone, mucosal reconstruction promotes reepithelialization and may enhance long‐term patency of the neo‐ostium.^15^ Moreover, mucosal preservation may improve patient‐reported olfactory function through sparing of olfactory fibers.^16^

Over the past decade, multiple single‐arm studies have reported high neo‐ostium patency rates following mucosal reconstruction across different flap designs.^10^, ^11^, ^12^ In 2019, Wang et al conducted the first and only randomized‐controlled trial investigating mucosal reconstruction after Draf III, demonstrating a reduction in stenosis with flap usage.^17^ Interestingly, previous systematic reviews and meta‐analyses stated no significant impact of the use of mucosal flaps/grafts in Draf IIb and Draf III.^7^, ^18^ However, these studies have included mucosal reconstruction as a subgroup and failed to control for heterogeneity in study design, patient selection, and surgical indications, limiting their validity.^7^, ^18^ Consequently, a comprehensive methodological analysis of the current evidence on mucosal flaps/grafts on neo‐ostium patency remains lacking.^19^, ^20^

This study aimed to evaluate the role of mucosal reconstruction in extended frontal sinus procedures by summarizing the evidence and conducting a meta‐analysis on its impact on neo‐ostium patency. By analyzing available studies, we seek to provide a clearer understanding of the role of mucosal flaps/grafts in frontal sinus surgery and identify areas for further investigation.

## Materials and Methods

### Search Strategy

A literature search was conducted according to Preferred Reporting Items for Systematic Reviews and Meta‐analyses (PRISMA) guidelines.^21^ Inclusion criteria were defined according to the PICO (patients, intervention, comparison, outcomes) format.^22^ PubMed, Medline, Cochrane, Scopus, and Web of Science were searched from inception to February 17, 2025. For search words, Medical Subject Headings (MeSH), controlled vocabulary, keywords, and adapted to the respective literature bank were used. The detailed search strategy is outlined in Supplemental Table S1, available online.

### Study Selection

Two authors (A.L., F.F.B.) independently screened abstracts and titles for relevant articles, followed by a full‐text review. Conflicting decisions on article inclusion were resolved through a third reviewer (D.T.L.) to reach consensus. Inclusion criteria were as follows: (1) patients with any frontal sinus disease; (2) patients undergoing Draf IIb or Draf III with mucosal graft or flap to potentially enhance patency; (3) documentation of subjective outcomes and/or objective outcomes (neo‐ostium patency or revision rate); and (4) mean follow‐up ≥ 6 months. Articles were excluded if they were (1) non‐English articles; (2) nonhuman data; (3) overlapping data; (4) review article, letters, and case reports with ≤10 patients; (5) lack of extractable data; (6) flap for skull base reconstruction; and (7) high risk of bias as defined below.

### Data Extraction

The final studies were analyzed, and relevant data were compiled into a standardized extraction sheet. This sheet comprised study and patient characteristics (eg, study country, study period, number of patients, age at surgery, and etiology), details on surgical procedure (eg, flap design, complications, use of stents, and postoperative care), patency rates and, if available, outcome measures (eg, intraoperative and postoperative neo‐ostium size, polyp score, and assessment of olfactory function). The evidence level for each study was assessed based on the Oxford Centre for Evidence‐Based Medicine Levels (level of evidence [LoE]).^23^ If only pictures were available, data were extracted with PlotDigitizer (available at: https://plotdigitizer.com/app). When mean values were not available, they were calculated according to Wan et al^24^ depending on patient number, estimating a normal data distribution. If flap‐specific follow‐up was not reported, the follow‐up for the respective total cohort was used. Since most neo‐ostium closures occur in the first 24 months post‐surgery, we defined ≥24 months as adequate follow‐up.

### Risk of Bias

To assess risk of bias, the Newcastle‐Ottawa Scale (NOS) was independently completed by two authors (A.L., F.F.B.) and discrepancies were resolved over discussion with a third author (D.T.L.). For the assessment of single‐cohort studies, the NOS cutoff was adapted as previously described.^25^ Consequently, comparative studies with ≤6 points and single‐arm studies with ≤4 were deemed to have a high bias risk, and the authors (A.L., F.F.B., and D.T.L.) discussed exclusion of these studies.

### Statistical Analysis

All statistical analyses were performed in STATA (StataCorp. 2021. Stata Statistical Software: Release 18. StataCorp LLC.) and Microsoft Excel (Microsoft Corporation, 2018. Microsoft Excel, available at: https://office.microsoft.com/excel). A *P*‐value of ≤.05 was considered significant. Continuous variables like age and follow‐up time were pooled by the ratio of means. For the dichotomous outcome measure “neo‐ostium patency,” the risk ratio (relative risk [RR]) was pooled. Only comparative studies with a direct control group were included in the meta‐analysis to ensure methodological consistency and to avoid bias from imputed control rates. Single‐cohort studies were reviewed qualitatively in the systematic review and analyzed via proportional meta‐analysis. The meta‐analysis for comparative studies was performed using the random‐effects model. Heterogeneity was assessed using Cochran's *Q* statistic (*P* < .10), *I*² (>50%), and *τ*² (<0.05). Results were summarized in effect size (ES), RR, and 95% confidence intervals (CIs). Subgroup analysis and meta‐regression were not conducted due to high heterogeneity among study variables, making meaningful subgroup comparison unfeasible. Risk for publication bias was assessed via funnel plot asymmetry and Egger's test.

## Results

**Figure 1:**
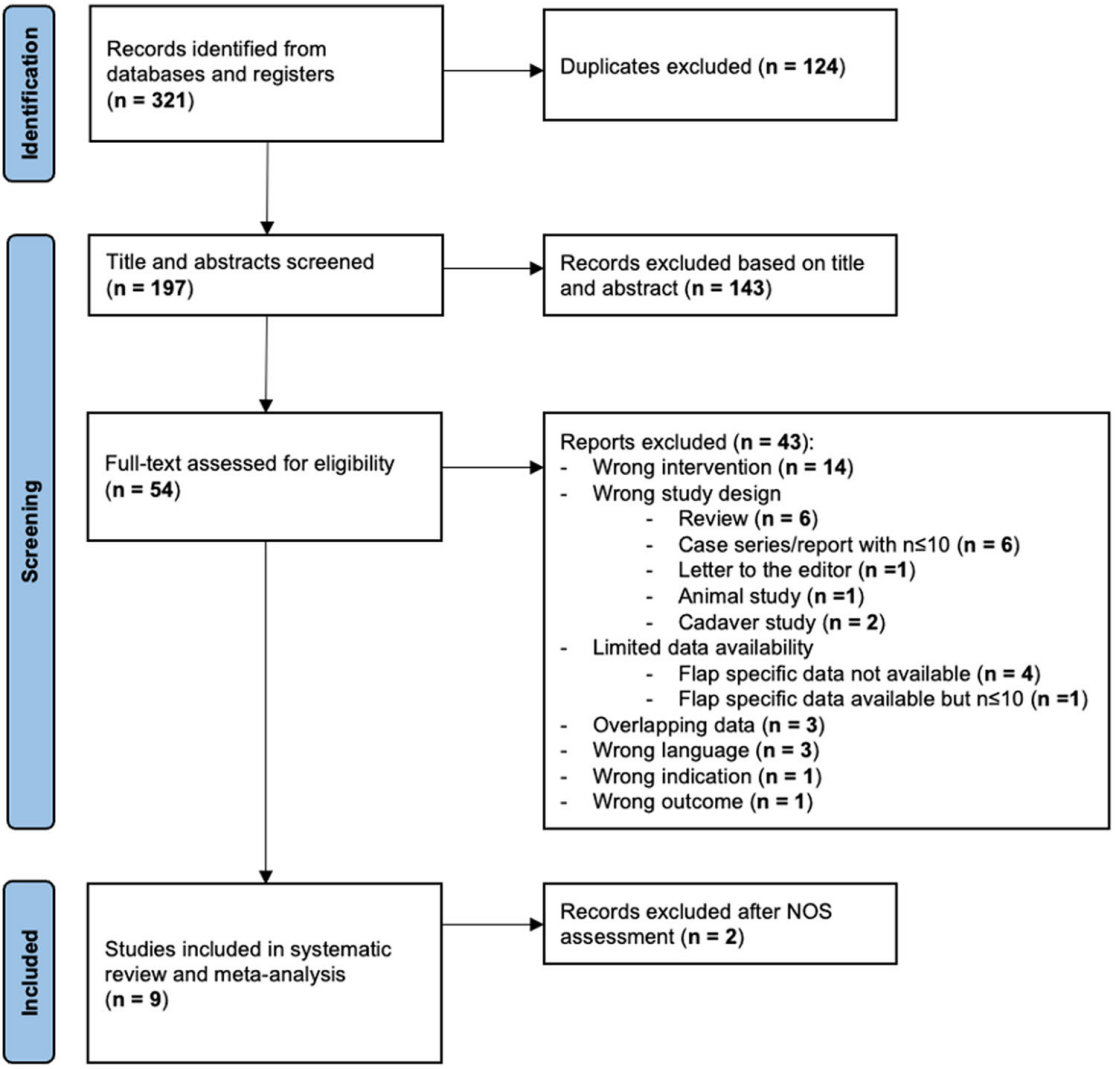
Literature search flow diagram according to the Preferred Reporting Items for Systematic Reviews and Meta‐analyses guidelines. NOS, Newcastle‐Ottawa Scale.

The results of the literature review are shown in Figure 1. Initial search resulted in 321 records (PubMed: 161; Scopus: 93; Web of Science: 59; Cochrane: 4; and Manual search of references: 4). Subsequently, studies were screened, whence 124 studies were excluded based on title, abstract, or if they were duplicates. Three studies^12^, ^26^, ^27^ were excluded due to the high risk of overlapping data. After NOS assessment, the studies by Wang et al^28^ and Khoueir et al^29^ were excluded due to scoring 6 points and 4 points, respectively, as well as both studies showing limited data availability (Supplemental Table S2, available online). Consequently, nine studies^11^, ^13^, ^14^, ^17^, ^30^, ^31^, ^32^, ^33^, ^34^ were included in the systematic review and meta‐analysis. For estimating the difference of neo‐ostium patency between patients with mucosal reconstruction technique and those without, we segregated the cohorts into “flap” and “no‐flap” cohorts. Patient characteristics, details on surgical procedure, and outcome measures were extracted from all nine studies.^11^, ^13^, ^14^, ^17^, ^30^, ^31^, ^32^, ^33^, ^34^

### Study and Patient Characteristics

Table 1 shows the study and patient characteristics of included studies. The nine included studies^11^, ^13^, ^14^, ^17^, ^30^, ^31^, ^32^, ^33^, ^34^ spanned 2006 to 2023 and were conducted in Germany (22%), Greece (11%), Taiwan/Australia (11%), China (22%), Italy (11%), United Kingdom (11%), and Japan (11%). The study designs included two retrospective case series,^11^, ^30^ two retrospective cohort studies,^14^, ^33^ four prospective cohort studies,^13^, ^31^, ^32^, ^34^ and one randomized‐controlled trial,^17^ with a LoE ranging from 3b to 1b. In total, 538 patients underwent frontal sinus surgery. Of those 6 (11.1%) patients underwent Draf IIa, 112 (21.9%) patients underwent Draf IIb, while 420 (78.1%) underwent Draf III. The patients' mean age was 50.1 (±4.9) years, including 346 (64.3%) males and 192 (35.7%) females. The primary indications for surgery were chronic rhinosinusitis (CRS) (n = 293), malignant or benign tumors (n = 99), mucocoele (n = 52), trauma (n = 22), and inverted papilloma (n = 30), with less frequent conditions such as encephalocoele (n = 7), cerebrospinal fluid (CSF) leaks (n = 9), osteoma (n = 9), and acute rhinosinusitis (n = 9). The mean follow‐up duration was 37.6 (±10.3) months.

**Table 1:** Study and Patient Characteristics

						Number of patients			Age					
First author, year	Country	Study period	Study type	LoE	Comparison	Total	Flap	No flap	Mean	(Range)	Gender	Procedure	Etiology	Follow‐up (mean)
Fischer, 2022	Germany	2012‐2021	Retrospective cohort study	3a	Flap versus no flap	123	86	37	52.6	18‐85	Female = 40, male = 83	Draf III	CRS (n = 69), encephalocoele (n = 5), trauma (n = 21), tumor (n = 28)	31.8
Leventi, 2024	Greece	2015‐2023	Retrospective cohort study	3a	Flap versus no flap	111	39	72	49	13‐78	Female = 46, male = 65	Draf III	ARS (n = 9), tumor (n = 43), CRS (n = 54), CSF (n = 5)	47
Wang, 2019	Taiwan/Australia	2014‐2018	Randomized control trial	1b	Flap versus no flap	50	27	23	48.4	NA	Female = 16, male = 34	Draf III	CRS (n = 45), mucocoele (n = 5)	34.8
Ye, 2023	China	2014‐2019	Prospective cohort study	2b	Flap versus no flap	43	20	23	47	19‐63	Female = 12, male = 31	Draf IIb (n = 9), Draf III (n = 34)	CRS (n = 22), mucocoele (n = 6), osteoma (n = 4), inverted papilloma (n = 11)	51.6
Fiorini, 2016	Italy	2010‐2014	Prospective cohort study	3b	Flap	46	46	‐	52	20‐84	Female = 10, male = 36	Draf IIb	CRS (n = 12), encephalocoele (n = 2), mucocele (n = 24), osteoma (n = 4), inverted papilloma (n = 4)	21^a^
He, 2022	China	2013‐2021	Prospective cohort study	3b	Flap	49	49	‐	48	19‐83	Female = 20, male = 29	Draf IIb	CRS (n = 32), mucocoele (n = 8), inverted papilloma (n = 9)	48.5^a^
Hildenbrand, 2021	Germany	2006‐2008	Retrospective case series	3b	Flap	24^b^	16	‐	43.8	20‐83	Female = 13, male = 11	Draf III	CRS (n = 9), mucocoele (n = 5), osteoma (n = 1), inverted papilloma (n = 1)	25.6
Illing, 2016	United Kingdom	2008‐2014	Prospective cohort study	3b	Flap	67	67	‐	54	15‐84	Female = 29, male = 38	Draf III	CRS (n = 37), tumor (n = 26), CSF (n = 4)	34
Omura, 2018	Japan	2013‐2018	Retrospective case series	3b	Flap	25	25	‐	62.3	36‐82	Female = 6, male = 19	Draf IIa (n = 6), Draf IIb (n = 9), Draf III (n = 11)	CRS (n = 13), tumor (n = 2), trauma (n = 1), mucocoele (n = 4), inverted papilloma (n = 5)	11.5

Abbreviations: ARS, acute rhinosinusitis; CRS, chronic rhinosinusitis; CSF, cerebrospinal fluid; LoE, level of evidence.

^a^ Approximated according to Wan et al.

^b^ Eight patients were lost to follow‐up; consequently, data on 16 patients were available for extraction.

### Surgical Procedures

**Figure 2:**
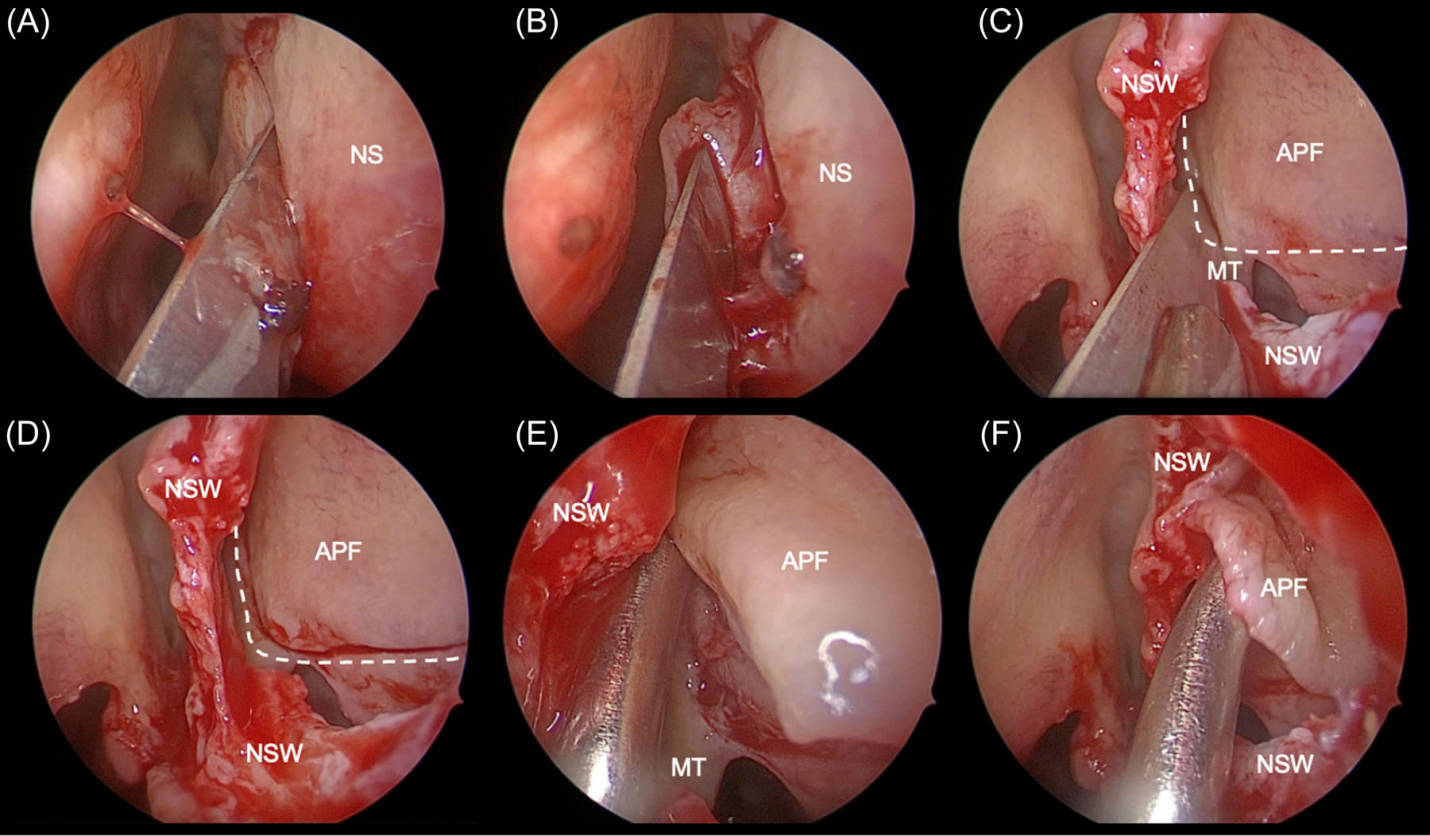
Anterior pedicled flap (APF) procedure.^17^ (A, B) Nasal septal window (NSW) creation. (C‐F) APF harvesting. Bilateral APFs cover lateral neo‐ostium; septal grafts covered frontal/posterior. MT, middle turbinate; NS, nasal septum.

Details on the surgical procedure are outlined in Table 2. Of 530 patients, 375 patients received mucosal reconstruction after 116 (30.9%) Draf IIb and 259 (69.1%) Draf III surgeries. Five studies^13^, ^30^, ^32^, ^33^, ^34^ used pedicle flaps, whereas mucosal grafts were used in one study.^11^ Two studies^14^, ^31^ allowed for either pedicled flaps or mucosal grafts, and one study^17^ used a combination of both. Flap designs were highly variable as shown in Table 2. Figure 2 shows an example of harvesting an anteriorly pedicled lateral nasal wall flap by one of the senior authors (F.F.B.). Exact rate of previous sinus surgery was reported in five studies^1^, ^11^, ^13^, ^14^, ^32^, ^33^ resulting in a 54.5% rate. However, one study^17^ reported only average rates of n = 2.3 previous surgeries, and one study^34^ only indicated a rate above 50%. Three studies^13^, ^30^, ^34^ explicitly reported no major complications, except one study^32^ noting four cases of nonobstructive synechiae. Only one study^17^ did not report the use of stents; however, stent design and postoperative aftercare protocols varied widely across studies and are outlined in Table 2. Two studies reported the use of postoperative antibiotics.

**Table 2:** Details on Surgical Procedure and Postoperative Care

First author, year	Flap type	Flap design	Previous sinus surgery	Delineation of surgical procedure	Complications	Use of stent	Aftercare	Antibiotics
Fischer, 2022	Pedicled flap	LPF	87/123	Yes	NA	Gelatin foam	NA	NA
Leventi, 2024	Pedicled flap or mucosal grafts	STF/LNSF/MG	71/111^a^	Yes	NA	Silicon roll	Nasal irrigation + nasal corticosteroids for patients with CRS	NA
Wang, 2019	Pedicled flap + mucosal grafts	MG + APF	2.3 (mean)	Yes	NA	NA	Tape occlusion of the nose for 2 wk Day 14: cleaning of nose + saline NaDo 2x per day	Co‐AmoxiClav for every patient for 7 d + 100 mcg mometasone 1x daily until 3 mo postop
Ye, 2023	Pedicled flap	LPF + LNSF + SF	33/53	Yes	0	Rubber finger stalk	Nasal saline irrigation + corticosteroids + decrusting	NA
Fiorini, 2016	Pedicled flap	STF	>50%	Yes	0	Silicone roll	NaDo	NA
He, 2022	Pedicled flap	LIPF	49/49	Yes	Nonobstructive synechia (n = 4)	Nasopore	Nasal saline irrigation 3x per day for 1 mo + budesonide nasal nebulization for recalcitrant CRS	NA
Hildenbrand, 2021	Mucosal graft	MG	1/16^b^	Yes	NA	Silicone roll	Tape occlusion of the nose to prevent crusting Day 7: if crusts are there, were left alone Day 14: cleaning of nose + start of Emser NaDo 2‐3x per day for 1 mo	For 5 d if nasal polyps: +50 mg prednisolone for 2 wk
Illing, 2016	Mucosal graft or pedicled flap^c^	MG/SF	NA	Yes	NA	Silicone sinus stent	NA	NA
Omura, 2018	Pedicled flap	LPF	NA	Yes	0	Nasal package with Sorbsan (calcium alginate)	NA	NA

Abbreviations: APF, anterior pedicled flap; CRS, chronic rhinosinusitis; LIPF, lateral inferior pedicle flap; LNSF, lateral nasoseptal flap; LPF, lateral pedicled flap; MG, mucosal graft; NaDo, nasal douche; SF, septal flap; SLAP, superior lateral anterior pedicle flap; STF, septoturbinal flap.

^a^ Reported only for the total cohort.

^b^ Previous sinus surgery is only reported on the patient who received revision surgery.

^c^ If posterior table cerebrospinal fluid leak was present, nasoseptal or free mucosal grafts were used.

### Patency Rates and Meta‐analysis

**Figure 3:**
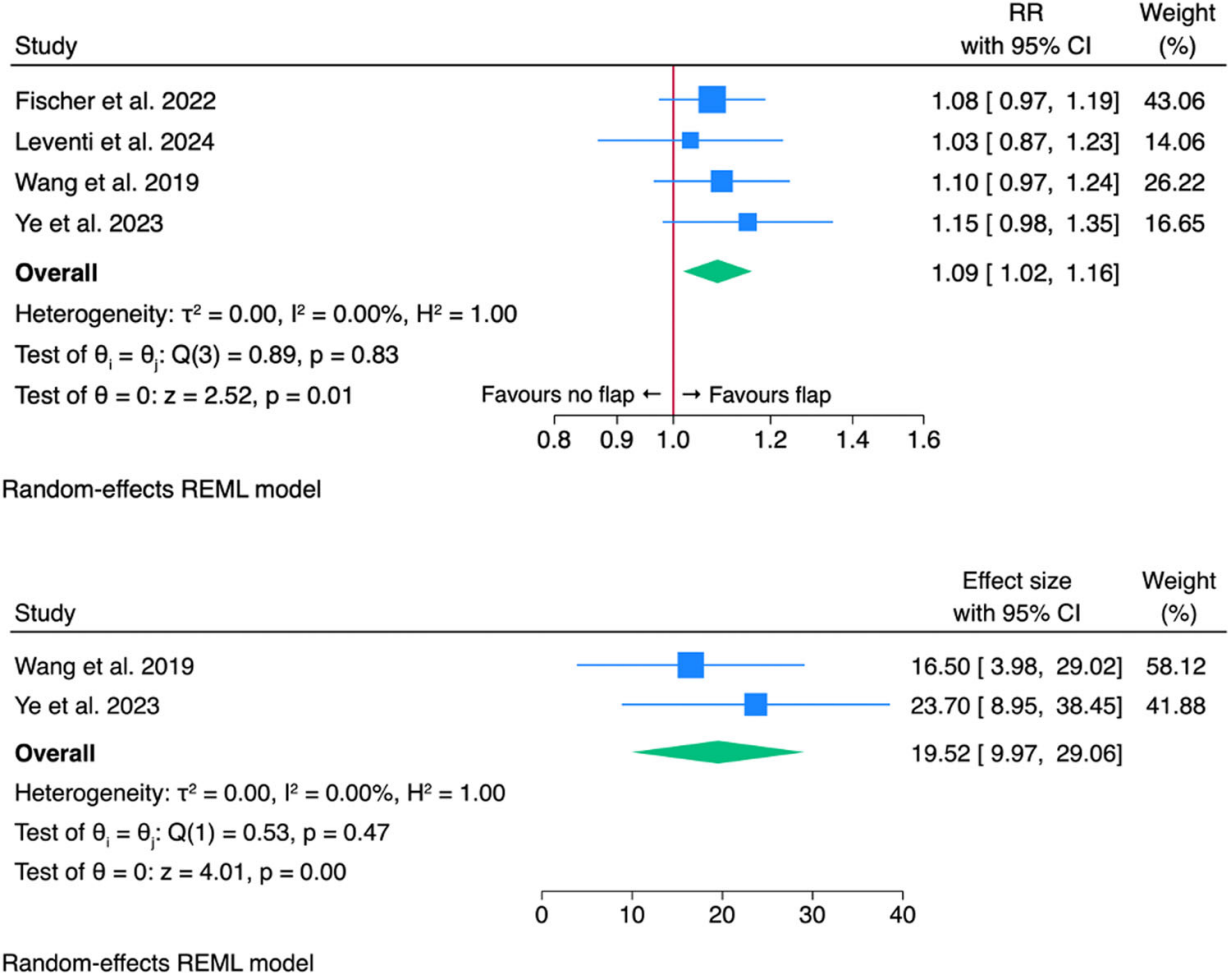
Forest plots of comparative studies regarding neo‐ostium patency and postoperative neo‐ostium square mean change. CI, confidence interval; REML, restricted maximum likelihood; RR, risk ratio.

**Figure 4:**
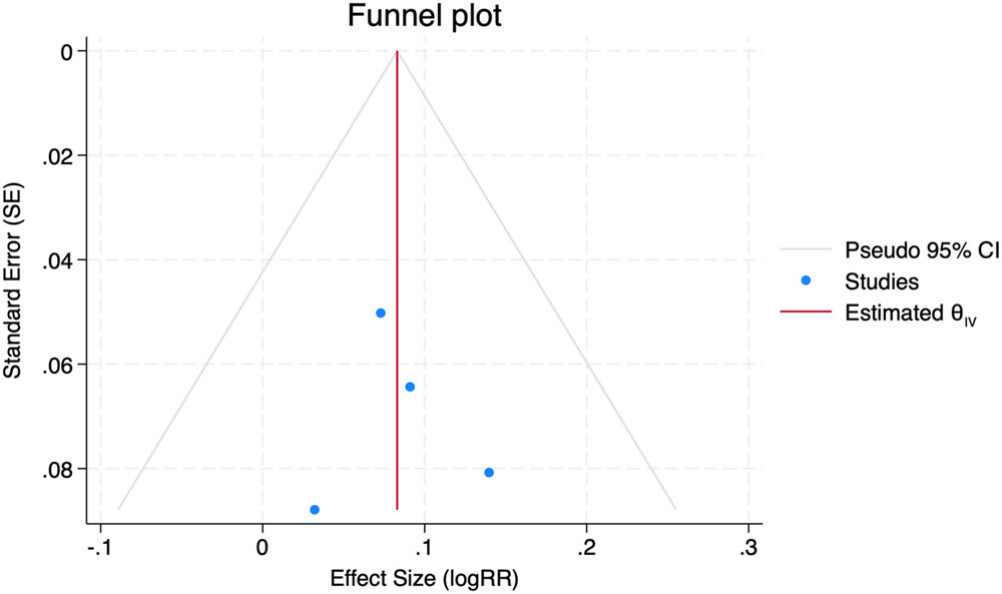
Funnel plot assessing publication bias. Blue dots: individual studies. Red vertical line: estimated overall effect size (*θ*_iv_). Gray triangular line: 95% CI. CI, confidence interval; RR, risk ratio.

Table 3 shows patency rates and definitions of failure. The definition of clinical failure varied across studies, with some defining it as total closure, whereas others considered the need for revision surgery or a reduction in recess size exceeding 50%. Over all nine studies,^11^, ^13^, ^14^, ^17^, ^30^, ^31^, ^32^, ^33^, ^34^ patency rate for the flap cohort was 96.1% (±5.2%) for the flap and 88% (±4.7%) for the no‐flap cohort. The meta‐analysis of comparative studies^13^, ^14^, ^17^, ^33^ included 327 patients, with 172 (52.6%) and 155 (47.4%) in the flap and no‐flap cohort, respectively. The overall mean age was 48.9 (±3.7) years. Consequently, the mean age was 49.3 (±3.0) years in the flap group and 48.6 (±3.7) years in the no‐flap group. For both cohorts, CRS was the most common etiology. Encephalocoele cases were exclusive to the flap group, while CSF leaks were only reported in the no‐flap cohort. Draf III was performed in nearly all cases (n = 318, 97.2%), with 165 (95.9%) in the flap group and 153 (98.7%) in the no‐flap group. The mean follow‐up was 41.3 (±9.5) months. Five studies reported A‐P distance and square mean of the neo‐ostium (Table 4). The meta‐analysis resulted in a pooled RR of 1.087 (95% CI: 1.018‐1.159, *P* = .011), indicating an 8.7% increase in neo‐ostium patency rate with mucosal flaps (Figure 3). Moreover, meta‐analysis revealed that the flap cohort exhibited a significantly greater mean postoperative neo‐ostium area compared to the no‐flap cohort, with a pooled mean difference of 19.52% (95% CI: 9.97‐29.06, *P* < .01). We observed no significant heterogeneity between studies (*τ*² = 0.00, *I*² = 0.00%, *H*² = 1.00, *P* = .83; *τ*² = 0.00, *I*² = 0.00%, *H*² = 1.00, *P* = .47). The funnel plot (Figure 4) appeared symmetric, suggesting no strong evidence of publication bias. This was confirmed by Egger's test (*P* = .92).

**Table 3:** Patency Outcomes

		Flap cohort				No‐flap cohort			
First author, year	Definition of clinical failure	Number of patients	Number of patent neo‐ostium	Patency rate, %	Revision surgery	Number of patients	Number of patent neo‐ostium	Patency rate, %	Revision surgery
Fischer, 2022	Total closure^a^	86	85	98.80	1/86	37	34	91.90	3/37
Leventi, 2024	Total closure	39	33	83.80	6/39	72	59	81.80	13/72
Wang, 2019	If revision surgery is required	27	27	100	0/27^b^	23	21	91.30	2/23
Ye, 2023	If revision surgery is required	20	20	100	0/20	23	20	87	3/23
Fiorini, 2016	If revision surgery is required	46	43	93.50	3/46	‐	‐	‐	‐
He, 2022	Total closure or need for revision	49	48	98	1/49	‐	‐	‐	‐
Hildenbrand, 2021	If revision surgery is required	16	15	94	1/16	‐	‐	‐	‐
Illing, 2016	A‐P reduction >50%^c^ + need for reoperation secondary to stenosis	67	65	97	2/67	‐	‐	‐	‐
Omura, 2018	Total closure	25	25	100	0/25	‐	‐	‐	‐

Abbreviation: A‐P, anterior‐posterior diameter.

^a^ Authors also reported “near total closure,” which was defined by not passable with a 5‐mm curved suction tip.

^b^ Reported three graft losses.

^c^ In total, 3/67 showed >50% reduction; however, of those, only two patients showed clinical failure and required revision surgery.

**Table 4:** A‐P Distance and Square Mean

		Flap								No‐flap cohort							
		A‐P distance				Square mean				A‐P distance				Square mean			
First author, year	Measurement	Intra‐OP	(±SD)	Post‐OP	(±SD)	Intra‐OP	Post‐OP	(±SD)	Change	Intra‐OP	(±SD)	Post‐OP	(±SD)	Intra‐OP	Post‐OP	(±SD)	Change
Fischer, 2022	Endoscopy	NA				NA				NA							
Leventi, 2024	Endoscopy	NA				NA				NA							
Wang, 2019	Endoscopy	15.1 mm	2.1 mm	12.7 mm	1.9 mm	100%	72.6%	16.10%	27%	14.3 mm	2.4 mm	10.5 mm	3.5 mm	100%	56.1%	26.80%	44%
Ye, 2023	CT	NA				2.76 cm^2^	2.05 cm^2^	0.73 cm^2^	−0.7 cm^2^	NA				3.3 cm^2^	1.67 cm^2^	0.74 cm^2^	−1.63 cm^2^
Fiorini, 2016	Endoscopy	NA				NA				NA							
He, 2022	Endoscopy	NA				NA				NA							
Hildenbrand, 2021	Endoscopy	NA				12.5 × 20.5 mm	9.6 x 15 mm	NA	36.90%	NA							
Illing, 2016	Endoscopy	11 mm	1.9 mm	9.9 mm	2.2 mm	100%	90%	NA	10%	NA							
Omura, 2018	CT	10.0 mm (median)	4.1‐16.3 mm (range)	9.6 mm (median)	6.3‐15.3 mm (range)	NA				NA							

Abbreviations: A‐P, anterior‐posterior diameter; CT, computed tomography.

### Clinical Outcomes

Overall, four studies^11^, ^13^, ^17^, ^31^ evaluated clinical outcomes (Table 5). Two studies^11^, ^17^ evaluated olfactory outcomes. Wang et al^17^ observed significant improvement, showing an improved score in the total cohort; however, no difference between groups. Hildenbrand et al^11^ showed that 13, 3, and 1 patients reported normal, impaired, and poor smelling, respectively. However, only postoperative values were assessed. Interestingly, Ye et al^13^ observed a significant decrease in osteitis assessed via global osteitis scoring scale in the flap cohort, whereas there was no significant change in the no‐flap cohort. Lund‐Mackay score and Lund‐Kennedy score were assessed by three^13^, ^17^, ^31^ and one^13^ study, respectively. Though initially planned, a meta‐analysis of clinical outcomes was omitted due to the great heterogeneity in reported clinical outcomes.

**Table 5:** Clinical Outcomes

First author, year	Lund‐Mackay score (LMS)	Lund‐Kennedy score (LKS)	Osteitis	Smelling outcomes	
Fischer, 2022	NA				
Leventi, 2024	NA				
Wang, 2019	Only pre‐OP LMS available (15.1 mean total cohort). No sig. difference between flap and no‐flap cohort (*P* = .81)	NA	NA	Significant improvement of TWSIT (pre‐OP 27.9 to post‐OP 31) for total cohort (*P* = .027). No significant difference between flap and no‐flap cohort (*P* = .92)	VAS improved from 4.4 to 3.1. (*P* = .063), with no difference between the flap and no‐flap cohorts (*P* = .85)
Ye, 2023	The flap and no‐flap cohort showed significant improvement (*P* < .001, *P* < .001). No comparison between cohorts was calculated	The flap and no‐flap cohorts showed significant improvement (*P* < .001, *P* < .001). No comparison between cohorts was calculated	Significant decrease in GOSS from 9.96 to 6.21 in the flap cohort (*P* = .01). No significant change in the no‐flap cohort (*P* = .46)		
Fiorini, 2016	NA				
He, 2022	NA				
Hildenbrand, 2021	NA	NA	NA	Only post‐OP values. 13, 3, and 1 patients report normal, impaired, and poor smelling, respectively	NA
Illing, 2016	Only pre‐OP values. No statistical test was performed	NA	NA	NA	NA
Omura, 2018	NA				

Abbreviations: GOSS, global osteitis scoring scale; TWSIT, the Taiwan Smell Identification Test; VAS, visual analog scale.

## Discussion

The present systematic review and meta‐analysis is the first detailed analysis of the available evidence on the use of mucosal flaps and grafts after Draf IIb and Draf III procedures. Mucosal reconstruction significantly improves the patency of the neo‐ostium, mostly in patients following Draf III surgery, and results in a greater neo‐ostium size. However, studies were highly heterogeneous regarding surgical procedure performed and outcome measures, omitting meaningful continuing synthesis.

### Patency Rate

The primary outcome of this meta‐analysis is that mucosal flaps and grafts significantly improve neo‐ostium patency rates. Combining all flap cohorts, we observed a pooled neo‐ostium patency rate of 96.1%, notably higher than the 83% patency rate reported in recent reviews for Draf III procedures.^1^ However, the use of flaps was not investigated in the noted study. For Draf IIb, the systematic review by Haddad et al^7^ reported a cumulative patency rate of 87.85%. The authors further analyzed the influence of flaps and grafts, observing a higher patency rate of 93.5%; however, this difference did not reach clinical significance. Another recent meta‐analysis comparing Draf IIb and Draf III procedures reported no significant benefit of mucosal flaps on neo‐ostium patency (odds ratio [OR]: 0.36, 95% CI: 0.09‐1.44, *P* = .15).^18^ However, this result was largely driven by the inclusion of Bastianelli et al^35^ who performed unilateral flap reconstruction and assessed healing time—not patency—as the primary outcome. This methodological mismatch led to its exclusion from our study. Additionally, the authors did not account for potential data overlap, which may have inflated the impact of high‐patency studies.^36^, ^37^ Another review on Draf III reported higher patency in the flap group but was limited by a follow‐up under 24 months and a small sample size (n = 80).^38^ In contrast, our analysis applied stricter inclusion criteria and focused on studies with defined patency outcomes and longer follow‐up, offering a more robust evaluation of mucosal flap efficacy.

### Follow‐Up

In the present study, the mean follow‐up duration of the included studies substantially exceeded 24 months. Naidoo et al reported that the neo‐ostium typically narrows within the first 24 months following Draf III procedures, followed by gradual widening—a process attributed to mucosal stabilization and thinning.^36^ This observation is consistent with findings by Ting et al, who noted that the majority of surgical failures (61%) occurred within the first 2 years.^39^ According to their analysis, early failure is primarily driven by osteoneogenesis and technical surgical factors, whereas late failure tends to reflect recurrence of the underlying disease. Similarly, Georgalas et al found that 60% of revision surgeries occurred within the first 2 years postoperatively.^40^ Consequently, these data suggest that the present meta‐analysis provides a reliable window to evaluate neo‐ostium stabilization. Nevertheless, follow‐up durations across the included studies in the present study ranged from 3 to 100 months; therefore, delayed failures may be underrepresented.

### Ostium Size

We observed a 20% less closure of neo‐ostium size for the flap cohort. As prior studies suggest, intraoperative neo‐ostium size correlates strongly with long‐term patency. For example, Ting et al reported a patency rate of 70.1% with a drill‐out size of 98 and 50 mm^2^ for bilateral and unilateral procedures, respectively.^39^ In contrast, Naidoo et al achieved a patency rate of 95% with a mean neo‐ostium size of nearly triple that—approximately 300 mm^2^.^36^ The authors hypothesized that larger ostia enhance topical drug delivery and provide greater tolerance for postoperative scarring.^36^, ^37^

In the present meta‐analysis, the mean reduction in neo‐ostium area was 25% for the flap cohort and 45% for the no‐flap cohort. However, the overall evidence on the extent of postoperative neo‐ostium area changes remains limited. Tran et al found a mean area loss of 25% at a 29.2‐month follow‐up, based on a baseline ostium size of 300 mm^2^.^6^ Similarly, Naidoo et al reported a 30% reduction within the first 2 years postoperatively. Interestingly, nearly 30% of patients in Tran's study experienced a ≥50% reduction in area within the first year, reflecting wide interindividual variability. Regarding flap use, only Ye et al and Wang et al provided comparative data on postoperative neo‐ostium dimensions, with mean sizes of 280 and 300 mm^2^, respectively.^13^, ^17^ These large ostia likely contributed to high‐patency rates, even in the absence of mucosal flaps. Future studies should investigate whether mucosal reconstruction improves patency in patients with smaller initial neo‐ostia or in those at higher risk for restenosis.

### Reduction of Inflammation

Heterogeneous outcomes across patients may be influenced by factors such as neo‐ostium size and the degree of local inflammation.^8^ Animal studies have shown that mucosal flaps can reduce histological markers of inflammation, suggesting a potential mechanism for improved healing.^15^ However, only one study in our analysis evaluated inflammatory parameters directly, limiting our ability to draw firm conclusions.

### Flap Design

A potential concern with free mucosal grafts is their susceptibility to displacement, particularly when loosely positioned over the neo‐ostium. Early postoperative nasal irrigation may dislodge the graft, compromising its integration and potentially reducing patency. However, this theoretical concern was not supported by the outcomes: Two studies employing free mucosal flaps reported high‐patency rates of 94% and 100%, respectively.^11^, ^17^ Both studies delayed irrigation and utilized nasal tape occlusion for 2 weeks postoperatively, potentially facilitating graft adherence and mucosalization. In contrast, Leventi et al was the only study to report immediate postoperative nasal irrigation in combination with free mucosal grafts, which may have contributed to its comparatively lower patency rate.^14^ Unfortunately, individual patency rates stratified by flap design were not reported, limiting the ability to draw definitive conclusions. Future studies should specifically examine the influence of postoperative care—particularly the timing of nasal irrigation—on graft survival and long‐term patency outcomes, especially in the context of free mucosal grafts. Standardized reporting of flap type and aftercare protocols is essential to better understand their contribution to surgical success.

### Olfactory Outcome

Although restenosis rate is important for the assessment of clinical failure, clinically relevant endpoints such as olfactory function or symptom improvement are even more important for patients. Preserving the mucosa of the superior septum or lateral nasal wall might play a crucial role in maintaining olfaction. In the present study, we observed only one study investigating olfactory function.^17^ Interestingly, there was no difference between the flap and no‐flap cohorts. This was also observed by Harvey et al who investigated olfactory function between patients with and without flap reconstruction through SNOT22.^40^ The authors revealed no difference between patients with flaps and those without.^41^ However, skull base reconstructions were mainly excluded from our study. To date, there is no clear evidence on the role of mucosal flaps on olfactory function.

### Limitations

The present meta‐analysis comes with several limitations. First, as with any meta‐analysis, inherent biases associated with the included studies may affect the overall findings. Second, relevant studies that have not explicitly mentioned mucosal reconstruction in their title or abstract were omitted. However, this approach was necessary to maintain a methodologically sound and reproducible search strategy. Third, we did not include studies that did not clearly define a frontal sinus procedure according to Draf IIb or Draf III. Multiple studies used flaps for skull base reconstruction, trauma closure or CSF closure together with the Draf III approach; however, these studies did not specifically examine the use of the flap to cover exposed bone. To eliminate this bias, we included only those studies that specifically analyzed the use of flaps. None of the included studies assessed radiological scores, inflammatory markers, and patient‐reported outcomes, such as olfaction, in combination. Finally, current literature is limited by considerable heterogeneity in flap design, surgical technique, postoperative care, and outcome reporting.

## Conclusion

Multiple studies have demonstrated that mucosal flaps and grafts are associated with significantly improved neo‐ostium patency; however, this could only be analyzed for Draf III. Consequently, prospective randomized trials are urgently needed to explore the role of flaps and grafts in Draf IIb surgery. Moreover, the impact of mucosal reconstruction on patient‐reported outcomes and olfactory function remains largely unexplored. Future research should incorporate standardized, multidimensional outcome measures to improve comparability and guide the selection of appropriate reconstructive techniques for specific clinical indications.

## Author Contributions

**Alexander Lein**, Writing—original draft, data curation, investigation, visualization, writing—review and editing; **David T. Liu**, Writing—original draft, investigation, writing—review and editing; **Birgit Knerer**, Data curation, investigation; **Christian A. Mueller**, Conceptualization, methodology, supervision, writing—review and editing; **Nicole Rotter**, Writing—review and editing, validation; **Erich Vyskocil**, Supervision, project administration; **Archana Jaiswal**, Formal analysis, methodology; **Faris F. Brkic**, Formal analysis, supervision, methodology, validation, writing—review and editing, project administration; **Rajiv Bhalla**, Conceptualization, supervision, writing—review and editing, project administration.

## Disclosures

### Competing interests

The authors declare no conflicts of interest regarding the present work.

### Funding source

No funding was received for this work.

## Supporting information

Supporting Information.
