# Supplementary material for: Mucosal Grafts and Flaps in Draf IIb and Draf III: A Systematic Review and Meta‐analysis
Source: Otolaryngol Head Neck Surg. 2025 Aug 4;173(5):1079–91. doi: 10.1002/ohn.1374 (PMC12574629; doi:10.1002/ohn.1374)
Supplement: Supplementary file 1 — Supporting Information. [file OHN-173-1079-s001.docx]

**Supplementary Tables**

| **PubMed:** |
| --- |
| <https://pubmed.ncbi.nlm.nih.gov/> |
| Search query: (“Lothrop” OR “frontal sinus drill-out” OR “frontal sinus drillout” OR “frontal sinus drillout*” OR “draf 3” OR “draf III” OR (“type III” AND “draf”) OR “draf 2b” OR “draf IIb” OR (“type IIb” AND “draf”) OR (“type 2b” AND “draf”)) AND (“flap” OR “mucosal flap” OR “graft*” OR “pedicled flap”) |
| è PubMed Screening resulted in 161 Titles and Abstracts |
| **Scopus:** |
| <https://www-scopus-com.ez.srv.meduniwien.ac.at/search/form.uri?display=advanced> |
| Search query: TITLE-ABS-KEY ( ( "Lothrop" OR "frontal sinus drill-out" OR "frontal sinus drillout" OR "frontal sinus drillout*" OR "draf 3" OR "draf III" OR ( "type III" AND "draf" ) OR "draf 2b" OR "draf IIb" OR ( "type IIb" AND "draf" ) OR ( "type 2b" AND "draf" ) ) AND ( "flap" OR "mucosal flap" OR "graft*" OR "pedicled flap" ) ) |
| è Scopus Screening resulted in 93 Titles and Abstracts |
| **Web of Science:** |
| <https://www-webofscience-com.ez.srv.meduniwien.ac.at/wos/woscc/advanced-search> |
| Search query (Used the combined feature for search queries): |
| AK=(( "Lothrop" OR "frontal sinus drill-out" OR "frontal sinus drillout" OR "frontal sinus drillout*" OR "draf 3" OR "draf III" OR ( "type III" AND "draf" ) OR "draf 2b" OR "draf IIb" OR ( "type IIb" AND "draf" ) OR ( "type 2b" AND "draf" ) ) AND ( "flap" OR "mucosal flap" OR "graft*" OR "pedicled flap" )) OR AB=(( "Lothrop" OR "frontal sinus drill-out" OR "frontal sinus drillout" OR "frontal sinus drillout*" OR "draf 3" OR "draf III" OR ( "type III" AND "draf" ) OR "draf 2b" OR "draf IIb" OR ( "type IIb" AND "draf" ) OR ( "type 2b" AND "draf" ) ) AND ( "flap" OR "mucosal flap" OR "graft*" OR "pedicled flap" )) OR TI=(( "Lothrop" OR "frontal sinus drill-out" OR "frontal sinus drillout" OR "frontal sinus drillout*" OR "draf 3" OR "draf III" OR ( "type III" AND "draf" ) OR "draf 2b" OR "draf IIb" OR ( "type IIb" AND "draf" ) OR ( "type 2b" AND "draf" ) ) AND ( "flap" OR "mucosal flap" OR "graft*" OR "pedicled flap" )) |
| è Web of science search yielded 59 results |
| **Cochrane library:** |
| <https://www.cochranelibrary.com/advanced-search> |
| Search query: (“Lothrop” OR “frontal sinus drill-out” OR “frontal sinus drillout” OR “frontal sinus drillout*” OR “draf 3” OR “draf III” OR (“type III” AND “draf”) OR “draf 2b” OR “draf IIb” OR (“type IIb” AND “draf”) OR (“type 2b” AND “draf”)) AND (“flap” OR “mucosal flap” OR “graft*” OR “pedicled flap”) |
| è Cochrane library search yielded 4 results |
| **Manual search of references:** |
| è Manual search yielded 4 results |

**Supplementary Table 1.** Search strategy for different databases.

| Study | Selection | | | | Comparability | Outcome | | | Total Score^a^ |
| --- | --- | --- | --- | --- | --- | --- | --- | --- | --- |
|  | Representativeness of exposed cohort | Selection of non-exposed cohort | Ascertainment of exposure^b^ | Outcome of interest not present at study start | Cohort comparability based on design or analysis^c^ | Assessment of outcome | Adequate lenght of follow-up for outcome?^d^ | Adequate cohort follow-up^e^ |  |
| Cohort Study Studys |  |  |  |  |  |  |  |  |  |
| Fischer et al. 2022 | * | * | * | * | ** | * | * | * | 9 |
| Leventi et al. 2024 | * | * | * | * | * | * | * | * | 8 |
| Wang et al. 2019 | * | * | * | * | ** | * | * | * | 9 |
| Wang et al. 2022 | - | * | * | * | * | * | - | * | 6 |
| Ye et al. 2023 | * | * | * | * | ** | * | * | * | 9 |
| Single Arm Studys |  |  |  |  |  |  |  |  |  |
| Fiorini et al. 2016 | * |  | * | * |  | * | - | * | 5 |
| He. et al. 2022 | * |  | * | * |  | * | * | * | 6 |
| Hildenbrand et al. 2021 | * |  | * | * |  | * | * | - | 5 |
| Illing et al. 2016 | * |  | * | * |  | * | * | * | 6 |
| Omura et al. 2018 | * |  | * | * |  | * | - | * | 5 |
| Khoueir et al. 2017 | - |  | * | * |  | * | - | * | 4 |

**Supplementary Table 2.** Results of the Newcastle-Ottawa Scale (NOS) for bias assessment

Asterix: **a**, A maximum of one star can be awarded for each NOS item, except comparability, which can be given a maximum of 2 stars; **b**, One star was awarded if mucosal flap positioning was reported in surgical records; **c**, One star was awarded if study adjusted for at least 1 key confounder. Two stars were awarded if study was adjusted for additional confounders; **d**, One star was awarded if follow-up was a minimum of 24 month post-operatively; **e**, One star was awarded if less then 25% of patient had missing follow-up measures. Changes for single-arm data: "selection of the non-exposed cohort" and "comparability of cohorts on the basis of the design or analysis" were not relevant for single-arm trials and were thus disregarded.
